# Repurposing ibuprofen-loaded microemulsion for the management of Alzheimer’s disease: evidence of potential intranasal brain targeting

**DOI:** 10.1080/10717544.2021.1937383

**Published:** 2021-06-12

**Authors:** Ming Ming Wen, Noha Ismail Khamis Ismail, Maha M. A. Nasra, Amal Hassan El-Kamel

**Affiliations:** a Department of Pharmaceutics & Pharmaceutical Technology, Pharos University in Alexandria, Alexandria, Egypt; b Department of Pharmaceutics, Alexandria University, Alexandria, Egypt

**Keywords:** Repurposing, ibuprofen, microemulsion, Alzheimer’s disease, intranasal delivery, brain targeting

## Abstract

Studies have shown the use of non-steroidal anti-inflammatory drugs, such as ibuprofen could reduce the risk of Alzheimer’s disease. The drug-repurposing strategy offers a bright opportunity for these patients. Intranasal administration through the olfactory pathway provides noninvasive and direct drug delivery to the target brain. A novel ibuprofen microemulsion was prepared, characterized and assessed the brain uptake in rats. The solubility of ibuprofen in various oils, surfactants, co-surfactants, and different ratios of surfactant/co-surfactant mixtures was screened and the phase diagrams were constructed. The colloidal particle size was 166.3 ± 2.55 nm and the zeta potential was −22.7 mV. Conductivity and dilution test identified an O/W type microemulsion with pH 4.09 ± 0.08. The rheological study showed a Newtonian flow behavior with cP 10.633 ± 0.603 (mPa⋅s). A steady drug release and linear permeation profiles were observed and showed a 90% permeation rate from the released drug. Ibuprofen microemulsion showed excellent stability in 3-months accelerated storage conditions, heating-cooling and freeze-thaw cycles, accelerated centrifugation, and 6- and 12-months long-term storage conditions. *In vivo* studies in rats further demonstrated a 4-fold higher brain uptake of ibuprofen from the microemulsion compared to the reference solution and nearly 4-fold and 10-fold higher compared to the intravenous and oral administrations. This study provides an exciting repurposing strategy and new administration route for the treatment of Alzheimer’s disease.

## 1. Introduction

‘Alzheimer’s is not just memory loss. Alzheimer’s kills’, the campaign slogan of the US Alzheimer’s Association indicated the cruelty of this disease. According to the 2019 Alzheimer’s disease (AD) facts and figures report, the number of deaths from AD stated on death certificates has increased by 145% between 2000 and 2017 in the US and the predicted number of AD patients will reach 14 million by 2050 if no new treatments are discovered (Alzheimer’s Association, 2019). Alzheimer’s Disease International also warned the global absence of a medical solution for AD and urged the need to find solutions by more AD research (Patterson, 2018). However, there is a slim chance to find more new therapies by 2025 (Cummings et al., 2016). Besides, more disappointing news was recently announced on failed phase III clinical trials for a new AD drug, aducanumab because of the uncertain risks (Eisai and Biogen, 2019).

As a result of the accumulation of beta-amyloid protein fragment outside the brain neurons, and the tau protein tangles inside the neurons, microglia are activated to remove these toxic proteins in the brains of AD patients (Hensley, 2010). When microglia cannot keep up with the rate of clearance needed, an inflammatory response occurs which may damage neurons and complicate the pathologic processes of the disease. Microglia and reactive astrocytes also activate the production of a wide range of pro-inflammatory genes that are associated with the death of neurons adjacent to the plaques (Mandrekar-Colucci & Landreth, 2012; McGeer et al., 2018). These shreds of evidence of neuroinflammation form a basic hypothesis that inflammatory mechanisms may play a role in AD and anti-inflammatory medications may reduce the risk of advancing AD by neuroprotection.

Several studies have found that the use of NSAIDs was associated with a decreased risk of AD. In a prospective, population-based cohort study in 6989 patients who did not have dementia at baseline showed the relative risk of AD fell to 0.20 with long-term NSAID use (in t’ Veld et al., 2001). Other studies also showed that NSAIDs inhibit β-amyloid aggregation (Combs et al., 2000), decrease cerebral Aβ burden and limit microglial and astrocytic activation (Ajmone-Cat et al., 2010), reduce AD risk, and slow disease progression (Szekely et al., 2008).

Being a disease of the brain, AD treatment presents an extreme and urgent challenge. Currently, the approved medications for AD are only symptomatic to improve memory loss temporarily and there are no available disease-modifying treatments (Alzheimer’s Association, Medications for Memory Loss, 2019). Over the years, there has been a lack of novel molecules entering into AD market. Therefore, the drug-repurposing strategy shifts research direction and offers a bright opportunity to find new uses for already approved drugs. For example, pioglitazone has been studied as repurposed management of AD (Jojo & Kuppusamy, 2019). In the search for novel therapies, drug repurposing can be an important novel treatment strategy for AD. The main advantage of the drug repurposing approach is that the safety issues have been proven and the drugs are already present on the market (Venkatachalam et al., 2019). Repurposing can be researched through in silico studies on the molecular level to predict and discover new therapeutic targets (Sohraby et al., 1903/2019) or studies of drugs that are relevant to a disease-modifying mechanism of action (Jojo & Kuppusamy, 2019). For the latter, the neuroinflammation mechanism in AD is a potential factor recommended to be taken into consideration for disease modification (Appleby et al., 2013).

Several NSAIDs, including ibuprofen, have been shown to reduce the risk of AD via the mechanisms independent of cyclooxygenase inhibition (Shoaib et al., 2017; Cole & Frautschy, 2012). Ibuprofen is a major medicine on the WHO model list of essential medicines and is available and used worldwide (World Health Organization (WHO), 2019). Epidemiological studies have shown that ibuprofen reduced the risk for neurodegenerative diseases such as AD and Parkinson’s disease (Dill et al., 2010). Through its effect on cyclooxygenase-1 suppression, ibuprofen may protect neurons against immune-mediated damage in the early stages of AD pathogenesis (Moore et al., 2010). Furthermore, ibuprofen has been exhibited improvement in cognitive dysfunction and histopathologic outcome in mouse models of AD (Sekiyama et al., 2012). It also reduces Aβ42 generation via inactivation of ras homolog gene family member A (RhoA) which subsequently stimulated axonal growth and promoted functional recovery in the damaged CNS rodent models (Zhou et al., 2003; Fu et al., 2007). These researches suggested the therapeutic potential of ibuprofen as a RhoA inhibitor in treating CNS injuries characterized by axonal disconnection. In general, NSAID therapy should be longer duration to affect the progression or the prevention of AD (Imbimbo et al., 2010). However, the long-term oral use of NSAIDs may cause gastrointestinal adverse effects due to COX-1 inhibition that should be considered especially in elderly patients with polypharmacy, and an alternative route of administration must be sought to avoid these side effects. One must also consider how to deliver sufficiently high concentrations of NSAIDs to the CNS, particularly the brain parenchyma for its use to be an effective medication against AD.

To overcome the limited blood-brain barrier (BBB) penetration, intranasal administration through olfactory delivery provides a rationale for a noninvasive alternative on the clinical ground to target the brain for direct drug delivery and offers benefit to avoid the side effects often associated with oral dosage forms of NSAIDs, such as gastrointestinal disturbances, gastric irritation, and increased risk of ulcer formation (Laine, 2003). In recent years, the olfactory mucosa has also been proposed as a potential target for an early marker of neurodegenerative conditions, such as schizophrenia, AD, multiple sclerosis, and Parkinson’s disease (Rey et al., 2018; Bhattamisra et al., 2020). Although olfactory epithelium is presented in only 3% of the nasal cavity, this route is short and direct because the olfactory sensory neurons do not have a synapse between the two-element sensory receptors (Wen, 2011). Ibuprofen has known limited ability to cross the BBB (Mandal et al., 2018); therefore, a novel formulation that can deliver ibuprofen through an intranasal route for brain targeting is vitally needed.

In this study, a novel ibuprofen microemulsion (ME) was formulated, characterized for repurposing use as a potential disease-modifying agent for the management of AD. The evidence of brain uptake through intranasal delivery was assessed for the potential application in direct brain delivery through the olfactory pathway.

## 2. Material and methods

### 2.1. Materials

Ibuprofen (Alexandria Co. for pharmaceuticals & chemical industries, Egypt.), oleic acid, soya bean oil and propylene glycol (Alpha Chemika, India), ethanol, polyethylene glycol 400 and glycerol (El Gomhouria, Egypt), miglyol, isopropyl myristate, Brij 35 and Labrasol (El Amriya, Egypt), castor oil, chamomile oil, clove oil, peppermint oil and jojoba oil (Chemajet, Egypt), Tween 20 and 80 (Oxford Lab Chem, India) and Transcutol (Gattefosse, France). All other chemicals were of analytical reagent grade.

### 2.2. Screening of microemulsion components and solubility study

‘Generally regarded as safe’ (GRAS) materials were carefully considered in the formulation of ibuprofen ME (FDA, 2018). Solubility of ibuprofen in various oils, surfactants, and co-surfactants was screened and the effect of different ratios of surfactant/co-surfactant mixtures (Smix) on ibuprofen solubilization capacity was also investigated to optimize the formulation. An excess amount of ibuprofen was placed in closed glass vials containing 5 ml of each tested component separately. The vials were then shaken in a thermostatically controlled orbital shaker (SW-20C, Julabo, Seelbach, Germany) at 60 rpm at 25 ± 0.5 °C for 3 days and left unshaken for another 24 h to reach equilibrium, following by centrifugation. The supernatant layer was separated to determine ibuprofen concentration by UV-Visible spectroscopy (Shimadzu 1800, Maryland, USA) at 264 nm. These tests were done in triplicate. Solubility study of ibuprofen in phosphate buffer solution (pH 7.4) was conducted in the same manner at 34 ± 0.5 °C to simulate the temperature in the nasal environment when calculating the sink condition in the drug release study.

### 2.3. Preparation of microemulsion

To prepare the ME, ibuprofen 400 mg was added to 2 g mixture of oil and *S*_mix_ and mixed till the drug was completely dissolved at room temperature. These mixtures were then gradually dropwise added with distilled water under stirring. Following the addition of each drop of water, the mixture was carefully examined for the formation of clear and transparent ME which was then equilibrated for 20 min and visually inspected for any other sign of physical change (Sintov & Botner, 2006). The amount of water added in each formulation was estimated, and the total weight of the preparation was determined to calculate the percentages of all three components.

### 2.4. Construction of pseudoternary phase diagrams

The selected oils, surfactants, and co-surfactants, according to the solubility studies were grouped in eight combinations and the phase diagrams constructed (SigmaPlot 14.5, Systat Software, Inc., San Jose, CA, USA) to indicate the stable area of the ME. To identify the limits of the one phase domain, a pseudoternary phase diagram was constructed with three axes representing aqueous, oil phase, and the mixture of surfactant and co-surfactant (*S*_mix_). The compositions of all studied pseudo ternary phase diagrams were presented in Table 1. In each formulation, a series of nine mixtures based on oil: *S*_mix_ volume ratios (1:9, 2:8, 3:7, 4:6, 5:5, 6:4, 7:3, 8:2, and 9:1) were prepared for the initial determination of ME phase areas. Extra points were also made to define the boundaries of phases when constructing the phase diagrams. Once the ME region was identified in each phase diagram, the largest ME areas with good physical stability were selected. Finally, a point in the middle of the ME region was identified for further characterization studies.

**Table 1.** Combinations of oils, surfactants, and co-surfactants used to construct the pseudoternary phase diagrams.

Formulation	Oil, *S_mix_ components	Weight ratio of *S_mix_
1	Oleic acid, Tween 20/ethanol	1:1
2	Oleic acid, Tween 80/ethanol	1:4
3	Oleic acid, Tween 20/propylene glycol	4:1
4	Oleic acid, Tween 80/propylene glycol	1:1
5	Soya bean oil, Tween 20/ethanol	1:1
6	Soya bean oil, Tween 80/ethanol	1:4
7	Soya bean oil, Tween 20/propylene glycol	4:1
8	Soya bean oil, Tween 80//propylene glycol	1:1

*S_mix_: surfactant/co-surfactant.

### 2.5. Drug content

Drug content was determined by measuring 100 µl of the selected ibuprofen formulations in 5 ml methanol. The samples were mixed and filtered through a 0.45 µm Millipore filter and assayed by UV-VIS spectrophotometry (Shimadzu UV-1800, Maryland, USA) at *λ*_max_ 264 nm. The measurements were performed in triplicate. Assay validation of the UV spectrophotometric assay was done in methanol and pH 7.4 phosphate buffer according to the International Conference on Harmonization (ICH) guideline for the parameters of linearity, accuracy, intra-day and inter-day precision, and reproducibility.

### 2.6. Colloidal particle size and zeta potential

Colloidal particle size, zeta potential, and polydispersity index (PDI) of the tested ibuprofen ME were measured using a dynamic light scattering instrument (Zetasizer NanoZS, Malvern Instruments, Worcestershire, UK). Measurements were performed in triplicates at 25 °C at a scattering angle of 173° using a glass cuvette with a square aperture. The measurement position was 4.65 mm and the dispersant refractive index was 1.33.

### 2.7. Conductivity measurement

The electrical conductivity of the ME was performed at 25 ± 2 °C using a conductometer TDS MI 170 (Martini instruments, Galliera Veneta, Italy) calibrated with a standard solution of KCl. The electrode was washed before each measurement, once using distilled water followed by absolute isopropanol and then with the sample twice. Three readings were taken in micro Siemens (µS). The device was calibrated using 1413 µS and 12,880 µS standards.

### 2.8. Dilution test

The dilution test was carried out by adding water and oil used in the formulation to determine the type of ME. Two vials of each tested formulation containing 10 ml of ibuprofen ME were prepared. To one vial, 2 ml of water was added and to another vial, 2 ml of oleic acid was added under continuous mixing at ambient temperature. The type of ME was determined according to the degree of dispersibility to the added water or oil as a continuous phase. If the ME becomes turbid upon dilution with water or oil, it was then defined as bicontinuous (Panapisal et al., 2012). The experiment was performed in triplicate.

### 2.9. pH measurement

The pH values of the tested formulations were determined in triplicates using a digital pH meter (Mettler Toledo, Scgott Geräte, Germany) standardized with pH 4 and pH 7 buffers before use.

### 2.10. Viscosity study

Tested ME of 8 ml was carefully introduced into a small sample adaptor chamber of the viscometer (Brookfield RV DV-II + pro, Middleboro, MA, USA) to avoid any entrapped air bubbles. Then a spindle (S15) was lowered into the chamber and rotated at speeds from 50 to 200 rpm for at least 2 min at each speed. Viscosity was measured at 25 ± 0.5 °C and calculated as a mean value of three measurements in centipoise (cP).

### 2.11. Transmission electron microscopy (TEM)

A drop of the tested ME was diluted with distilled water and placed on a carbon-coated copper grid with filter paper, followed by placing a drop of uranyl acetate on the sample for staining and then wiped off. The surface of the carbon grid was let air-dried before being loaded onto the transmission electron microscope (Joel JEM 1230, Tokyo, Japan).

### 2.12. *In vitro* drug release study

In vitro release study was carried out using modified Franz diffusion cells (Nanseng Lab Glass, Taipei, Taiwan) which consisted of a receptor compartment (21 ml) and a donor compartment with an opening diffusion area of 4.52 cm^2^ (Wen et al., 2012). An outer layer of glass jacket surrounded the receptor chamber to allow an inflow of water to the jacket from a circulating water bath to maintain the temperature of diffusion cells at 34 ± 0.5 °C which was the temperature of the nasal cavity (Zhang et al., 2010). The receptor compartment was filled up with pre-warmed phosphate buffer (pH 7.4) and stirred throughout the experiment to ensure sink conditions. Cellophane membrane (12–14 kDa cutoffs, Spectra/pro® Spectrum Laboratories Inc., California, USA), presoaked in receptor medium overnight, was mounted on the diffusions cells between the donor and receptor chambers. The diffusion cell sets were secured with clamps and left to equilibrate with the receptor medium for 30 min before the experiment.

ME containing 200 mg/ml ibuprofen or ibuprofen in propylene glycol solution (200 mg/ml), as a comparator, were placed in the donor chamber. At predetermined time intervals, 1 ml sample was withdrawn from the center of the receptor compartment, filtered, diluted, and assayed by UV-spectrophotometer at *λ*_max_ 264. The assay was linear in the ibuprofen concentration range of 0.15–0.4 mg/ml (*n* = 5, *y* = 1.4286*x* + 0.0209, *R*^2^ = 0.9996). The percentage recovery ranged from 93.18 to 101.883 and intra-day and inter-day precision with the CV% ranged from 0.05 to 5.73%. Removed samples were immediately replaced by equal volumes of pre-warmed receptor medium to maintain a constant volume in the receptor compartment throughout the experiment. This sample dilution factor was corrected by the DDSolver Add-In program (Zhang et al., 2010). Data were expressed as mean ± SD of triplicates.

### 2.13. *In vitro* nasal mucosa membrane permeation study

Permeation study was performed using freshly excised sheep nasal membrane in modified Franz diffusion cells same as reported above. The average thickness of the sheep nasal mucosal membrane was 0.32 ± 0.07 mm (*n* = 6). A piece of excised nasal mucosa membrane was mounted between the donor and receptor compartments with the mucosal layer facing upward to the donor side. The membrane was left for equilibrium with the receptor fluid for 30 min before the experiment. A volume of 100 µl tested ME was gently placed in the donor chamber. The receptor compartment was filled with 21 ml of phosphate buffer (pH 7.4) and constantly stirred at 34 ± 0.5 °C. All other experimental steps were the same as reported above. Each study was carried out in 6 h. Blank ME was used as a comparator for each measurement. Release flux, permeability coefficient (*K_P_*), lag time (*T_L_*), and diffusion coefficient (D) of ibuprofen were calculated based on the reported mathematic model (Mitragotri et al., 2011).

### 2.14. Stability study

#### 2.14.1. Accelerated stability study

The tested formulations were stored in a standard stability chamber at 40 °C and 60% relative humidity (RH) for 3 months. Samples were evaluated by visual examinations, drug content, particle size, pH, TEM, and in vitro drug release studies.

#### 2.14.2. Physical stability study

Three types of physical stability testing were examined namely, clarity, phase separation, and precipitation of ibuprofen ME following the below-mentioned tests. All tests were performed in triplicate. The viscosity of samples was also measured and compared to the fresh samples.

##### 2.14.2.1. Heating-cooling cycle test

Samples were stored in the refrigerator at 4 ± 1 °C for 2 days and then at 45 ± 1 °C in a hot air oven for another 2 days. Six heating and cooling cycles were repeatedly carried out (Shafiq-un-Nabi et al., 2007).

##### 2.14.2.2. Freeze-thaw cycle test

Samples were stored in the freezer at −21 ± 1 °C for 2 days and then at ambient temperature for another 2 days. Three cycles were repeated in this manner (Li et al., 2018).

##### 2.14.2.3. Accelerated centrifugation test

Samples were subjected to strong gravitational stress by centrifugation at 3500 rpm for 30 min at room temperature (Restu et al., 2015).

#### 2.14.3. Long term stability study

Samples were stored at room temperature and ambient humidity (40%) for 6 months and 1 year. Signs of drug precipitation, phase separation, and/or color change were regularly recorded. Drug content and pH measurements were also evaluated.

### 2.15. *In vivo* study

The in vivo experimental procedures followed the ARRIVE guidelines for animal study (Kilkenny et al., 2010). Sprague-Dawley rats (male, 300–350 g) were provided by the animal facility of the Faculty of Pharmacy, Pharos University in Alexandria. All rats were fed a standard commercial pellet diet and kept in well-ventilated houses with a controlled light-dark cycle at ambient temperature and 50% humidity. Rats were anesthetized by intraperitoneal injection of 0.02 ml ketamine (100 mg/ml) and 0.01 ml Xyla-Ject (20 mg/ml) before drug administration. The experimental protocol (PUACF-053-065) was approved by the Research Ethics Committee of Pharos University in Alexandria and the Institutional Animal Care and Use Committee (Faculty of Pharmacy, Alexandria University).

#### 2.15.1. Study design

Fifteen rats were divided into five groups, with three rats in each group. The first group was intranasally administered of the selected ME, the second group was intranasally administered ibuprofen in propylene glycol solution as a reference control, the third group received intranasal administration of blank ME, the fourth group was given oral administration ME, and the fifth group was intravenous administration of ME. All groups received 100 µl tested formulation, with 2.0 mg ibuprofen or blank. A nasal installation volume of 100 µl was considered as an acceptable delivery volume in rats (Zhou et al., 2020). The dose was calculated according to the lowest oral bioavailability dose in adult humans of 400 mg/tablet (in t’ Veld et al., 2001). The intranasal administration was delivered using a cannula needle tube connected to a micropipette, with a blunt end inserted 8 mm into one nostril and the other nostril was free to breathe. The tested sample was slowly pushed into the nasal cavities dropwise for 15 min. All rats in groups 1, 2, and 3 were in supine positions with the head elevated at 30 degrees for easier administration of the drug. The tube was removed after completion of administration, and the rats were kept immobilized for 30 min to avoid the loss of drugs.

#### 2.15.2. Brain homogenates preparation

All rats were sacrificed 1 h after the administration. Whole-brain organs were collected and weighed. For each brain organ, the equivalent weight of methanol was added and homogenized with a high-speed homogenizer (IKA T25 digital ultra- TURRAX, Staufen, Germany) at 10,000 rpm/min for 10 min. Two ml of the brain homogenate were then transferred into Eppendorf tubes, followed by the addition of 1 µl of ketoprofen (3 µg/ml) as an internal standard to give a final concentration of 0.0015 µg/ml. The mixture was centrifuged at 3000 rpm for 30 min. The supernatant was filtered through a 0.2 µm PTFE syringe filter (Santorious, Göttingen, Germany), and the amount of drug that reached the brain was assayed by HPLC.

### 2.16. HPLC analysis

HPLC (Agilent Technologies 1200 Infinity Series, Santa Clara, CA, USA) was equipped with an isocratic pump (G1310B), a variable wavelength detector (G1314F), and a manual injector (G1328C). Chromatographic separation was performed using a reversed-phase C18 column. The mobile phase consisted of 750 ml methanol, 3 ml phosphoric acid, and 247 ml water and was delivered at a flow rate of 1.5 ml/min at room temperature. The injection volume was 20 µl and the column effluent was detected at wavelength 264 nm.

#### 2.16.1. Preparation of brain homogenate calibration curve

Twenty-four rats were used to construct the calibration curve of ibuprofen in rat’s brain homogenate (British Pharmacopoeia Commission, 2013) which consisted of six concentrations in the range of 0.05–1 µg/ml. For each concentration point, four rats were sacrificed and their whole brains were harvested and weighed. An equivalent weight of ibuprofen standard solution was added to the brain sample. The mixture was then homogenized using a high-speed homogenizer at 10,000 rpm/min for 10 min. All the following procedures were the same as reported in 2.15.2. The calibration curve was constructed by plotting the peak area ratio of ibuprofen to ketoprofen against the concentration of ibuprofen in µg/ml.

#### 2.16.2. Validation of HPLC assay

Ibuprofen HPLC assay was validated according to the ICH guidelines (Center for Drug Evaluation and Research, 1995) to assure linearity, range, accuracy, intra-day and inter-day precision, and repeatability. The linearity of the brain homogenate calibration curve was determined by the serial dilution method over ibuprofen concentrations between 0.05 and 1 μg/ml. The percentage of ibuprofen recovery from the spiked standard solution samples was used to estimate the accuracy of the assay while the CV% was used to represent the inter-day and intra-day precision. Different calibration curves, performed on 3 different days, were tested for repeatability.

### 2.17. Nasal ciliotoxicity study

A nasal ciliotoxicity study was carried out using in vivo rat nasal mucosa model (Xie et al., 2006) with minor modification. Eight Sprague-Dawley rats (176–200 g) were divided into four groups with two rats in each group. Group 1 and 2 are negative and positive control rats that were administered with 100 µl saline and propranolol 2% w/v solution, respectively. The other two groups were given 100 µl blank and tested ME formulation containing 2.0 mg ibuprofen. All rats were administered via the intranasal route. Treatment was given once a day for 6 days. All rats were sacrificed 24 h after the last administration. The nasal mucosa was carefully separated and stored in 4S1G fixative solution, and examined by scanning electron microscope with software SEM control user interface, version 5.09 (JOEL, Tokyo, Japan).

### 2.18. Statistical analysis

Data were statistically analyzed by analysis of variance ANOVA using GraphPad Prism version 7.0 for Windows (GraphPad Software, La Jolla, California, USA). Post hoc multiple comparisons test was applied when necessary. *p* ≤ .05 indicated statistical significance while *p* ≤ .001 indicated highly statistically significant values.

## 3. Results and discussion

### 3.1. Solubility study

In the ME, ibuprofen might be solubilized in the oily core and/or on the interface between the surfactant and co-surfactant. Inevitably, the choice of the oil phase, as well as the surfactant and co-surfactant, has a marked impact on the drug solubility in the stable ME (Zhao et al., 2013). Table 2 showed the solubility data of ibuprofen in the screened ME components. Ibuprofen had the highest solubility in oleic acid (214.11 ± 0.22 mg/ml), followed by soya bean oil (122.43 ± 0.21 mg/ml) compared to other oils. This may be attributed to the polarity of the poorly soluble ibuprofen that favors its solubilization in small/medium molar volume oils, such as medium-chain triglycerides or mono- or di-glycerides (Lawrence & Rees, 2012). Edible oils such as castor oil, clove oil, and peppermint oil showed poor ability to dissolve large amounts of lipophilic ibuprofen.

**Table 2.** The solubility of ibuprofen in various tested microemulsion components at 25 °C (*n* = 3).

Microemulsion component	Type	Ibuprofen solubility (mg/ml)
Oil	Oleic acid	214.11 ± 0.22
	Soya bean oil	122.43 ± 0.21
	Castor oil	116.52 ± 1.29
	Miglyol	57.11 ± 2.05
	Chamomile oil	23.41 ± 0.05
	Clove oil	22.11 ± 0.81
	Isopropyl myristate	20.12 ± 0.03
	Peppermint oil	19.28 ± 0.02
	Jojoba oil	14.82 ± 0.01
Surfactant	Tween 80	370.82 ± 0.02
	Tween 20	302.45 ± 0.01
	Labrasol	254.66 ± 0.02
	Brij 35	64.68 ± 0.01
	Sorbitol	6.86 ± 0.01
	Transcutol	4.32 ± 0.00
Co-surfactant	Ethanol	735.54 ± 0.06
	Propylene Glycol	469.07 ± 0.03
	PEG 400	302.45 ± 0.02
	Glycerol	5.64 ± 0.00
Surfactant\Co-surfactant	Tween 20\Ethanol	(1:1) 1035.10 ± 0.17
		(1:4) 1019.39 ± 0.15
		(4:1) 818.49 ± 0.02
	Tween 20\Propylene glycol	(4:1) 995.4 ± 0.09
		(1:1) 509.84 ± 0.03
		(1:4) 19.63 ± 0.00
	Tween 80\Ethanol	(1:4) 1113.3 ± 0.05
		(4:1) 613.74 ± 0.06
		(1:1) 425.7 ± 0.02
	Tween 80\Propylene glycol	(1:1) 763.71 ± 0.07
		(1:4) 567.46 ± 0.01
		(4:1) 545.22 ± 0.3

In surfactants, higher ibuprofen solubility was observed in Tween 80 and Tween 20 with 370.82 ± 0.018 mg/ml and 302.45 ± 0.018 mg/ml, respectively. Ibuprofen had higher solubility in Tween 80, Tween 20, and Labrasol compared to all tested oils. The required surfactant HLB value to form o/w microemulsion is usually greater than 10 (Kommuru et al., 2001), and Tween 80 and Tween 20 has HLB of 15 and 16.7. These hydrophilic surfactants with higher HLB values prefer the interface in the formation of ME to lower the energy required, consequently improving the stability. Other reasons to consider using nonionic surfactants include that they are less affected by pH changes in ionic strength, relatively less toxic than ionic type surfactants, and generally have lower critical micelle concentrations (Sheskey et al., 2020).

The use of a single surfactant may not achieve transient negative interfacial tension and fluid interfacial film during the formation of ME; therefore, co-surfactants are often added to support the structure of ME at low surfactant concentration by reducing the interfacial tension. Co-surfactants also increase the mobility of the hydrocarbon tails of surfactant to allow greater penetration of the oil into this region (Alany et al., 2000). Their use also decreases the bending stress of the interface; therefore, creating a more flexible interfacial film that can take up different curvatures required to form ME over a wide range of compositions (Swarbrick, 2013). For example, ethanol not only increases the miscibility of the aqueous and oil phases by placing itself among surfactant heads, resulting in a higher dielectric constant but also decreases the mixture viscosity, making it easier for the Tweens to reach the interface faster (Reekmans et al., 1990). Ibuprofen showed the highest solubility in ethanol and propylene glycol with 735.54 ± 0.061 mg/ml and 469.07 ± 0.032 mg/ml, respectively, compared to PEG 400 of 302.45 ± 0.02 mg/ml. In comparison with ethanol and propylene glycol, PEG 400 may increase the risk of destroying the ME system due to its relatively higher hydrophilicity (Prieto & Calvo, 2013). Remarkable improvement of ibuprofen solubility was noted in the combination of surfactant and co-surfactant in different ratios which suggested that the optimal ratios for Tween 20 were 1:1 in ethanol and 4:1 in propylene glycol; and 1:4 in ethanol and 1:1 in propylene glycol for Tween 80.

### 3.2. Construction of pseudo-ternary phase diagrams

ME is a thermodynamic stable system due to its low inner energy state and reduced interfacial tension at the oil-water interface and usually forms within specific concentration ranges of components. It has the benefit of low production cost. To reach the thermodynamic stable system, the formulation requires optimization of the oil, surfactant/co-surfactant, and aqueous components. Therefore, the pseudo-ternary phase diagrams were established to identify the optimized ME regions and understand the complex interaction that could occur when different components were mixed. In the phase diagram, each corner represented 100% of the water, oil, and the mixture of surfactant/co-surfactant. A clear, transparent, and the fluid system was identified as ME.

**Figure 1.**
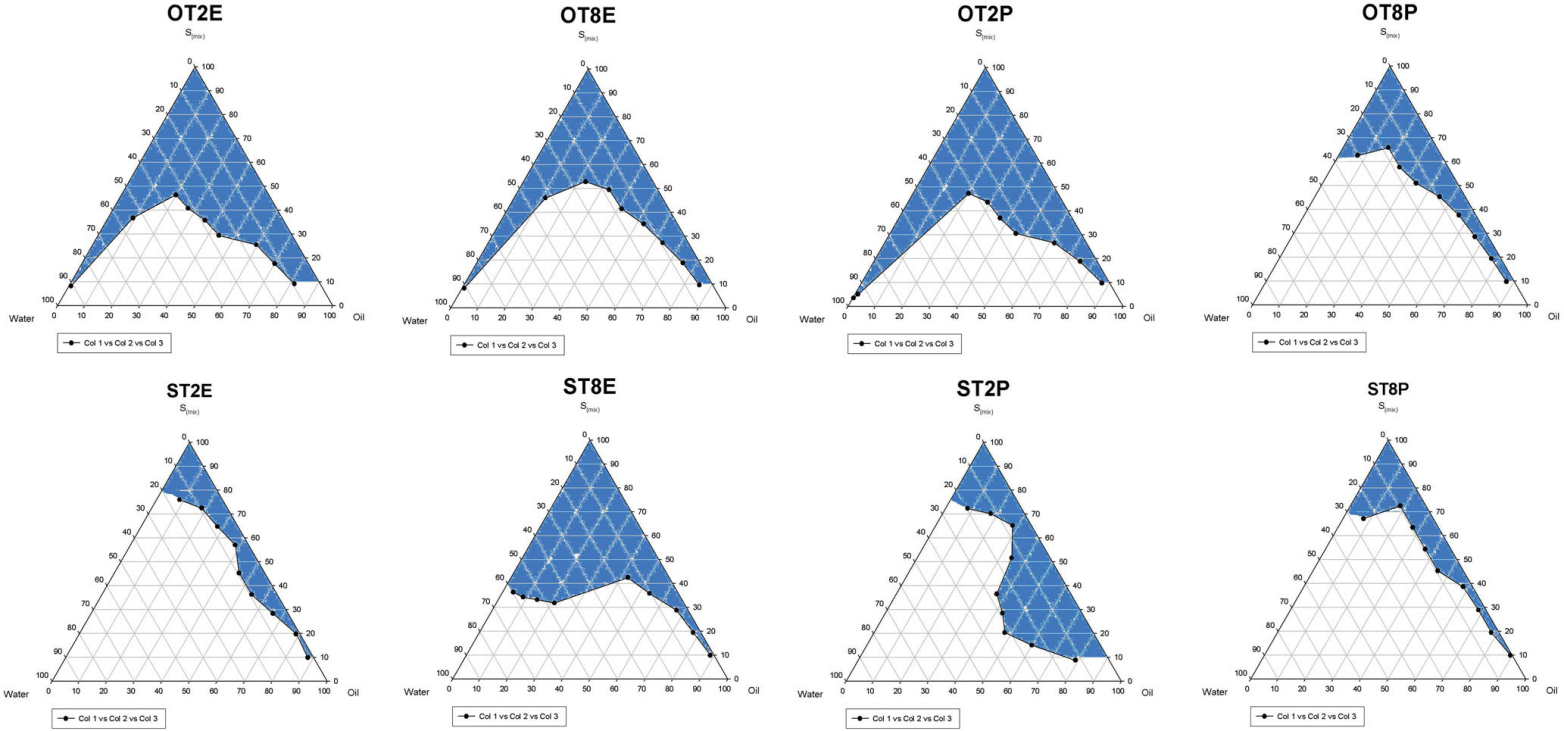
Pseudoternary phase diagrams of the tested microemulsion formulations.

Based on the solubility study results, oleic acid, soya bean oil, Tween 80, Tween 20, propylene glycol, ethanol, and distilled water were included in the ME formulation. These components were grouped into eight different combinations for phase studies (Table 1). Pseudoternary phase diagram was constructed for each group to pinpoint the ME area that was shaded for clarification (Figure 1). Outside the shaded area indicated turbid regions or regions where phase separation was observed.

The phase diagrams indicated that the ME area increased with an increase in the weight ratio of surfactant to co-surfactant. F1, F2, F3, and F6 showed larger ME zones compared to other formulations. F3 and F6 were excluded from further investigations due to visible phase separation and drug precipitation upon storage that could be resulting from the lower ibuprofen solubility in soya bean oil (122.43 ± 0.21 mg/ml) and *S*_mix_ of Tween20/propylene glycol (4:1, 995.4 ± 0.09 mg/ml) compared to oleic acid (214.11 ± 0.22 mg/ml) and *S*_mix_ of Tween 20/ethanol (1:1, 1035.10 ± 0.17 mg/ml), respectively. On the other hand, the high HLB values of Tween 20 and Tween 80 in F1 and F2 enabled highly polar oil such as oleic acid to be localized at the interface to form stable ME. Therefore, F1 and F2 were selected to continue further investigations. The middle points of the ME regions in F1 and F2 phase diagrams were identified to avoid the metastable area and the content ratios were located as 2:2:6 (oleic acid: water: Tween 20/Ethanol) in F1 and 2:2:6 (oleic acid: water: Tween 80/Ethanol) in F2.

### 3.3. Drug content recovery

Ibuprofen content recovery in F1 and F2 were 98.7 ± 3.6% and 99.5 ± 3.9%, respectively. There was no significant difference in drug content between these freshly prepared two formulations (ANOVA, *p* > .05).

### 3.4. Colloidal particle size, polydispersibility index, and zeta potential

Table 3 showed the colloidal particle size, zeta potential, and PDI of F1 and F2. The average droplet sizes were 166.3 ± 2.55 nm (F1) and 84.13 ± 4.75 nm (F2). A significant decrease in droplet size was observed in F2 containing Tween 80 compared to F1 containing Tween 20 (*p* ≤ .05). The reduction of droplet size led to the formation of more stable, isotropic ME and providing a larger surface area for more effective drug delivery. Both F1 and F2 were homogenous ME formulations with PDI 0.24 ± 0.3 and 0.23 ± 0.02, respectively. Zeta potential presented negatively charged globules of −22.7 mV in F1 and −41.1 mV in F2, indicating a prediction of the long-term ME stability (Patel et al., 2013). The resulting negative charged droplets might be due to a sudden expulsion of OH groups from the oil/water surface above given critical concentration of the surfactant and the presence of free fatty acids as impurities that commonly exist in nonionic surfactants (Manev & Pugh, 1991). F2 had a larger negatively charged zeta potential compared to F1. It may be associated with the higher emulsification power of Tween 80/ethanol (1:4) than Tween 20/ethanol (1:1), resulting in a decrease in surface tension and surface free energy of the micelles (Patel et al., 2013). Furthermore, compared to Tween 20, Tween 80 has a higher negative isothermal variation of the entropy (Δ*S_t_*) and is composed of a longer hydrocarbon chain which contributes to its lower critical micelle concentration (Kerwin, 2008). The presence of a higher amount of ethanol in F2 may also cause a pronounced decrease in surface tension, and subsequently more negative zeta potential. It has been reported that ME was physically stable and less liable to phase separation when the zeta potential was around −30 mV (Li et al., 2002). Therefore, both F1 and F2 were considered promising ME formulations with well-defined globule sizes, zeta potential, and PDI.

**Table 3.** Colloidal particle size, polydispersity index, zeta potential, conductivity and pH data of F1 and F2 measured at 25 °C (*n* = 3).

Formulation	Colloidal particle size Mean ± SD (nm)	PDI ± SD	Zeta potential (mV)	Conductivity		pH
				Conductivity Mean ± SD (μS/cm)	CV%	
F1	166.3 ± 2.55	0.24 ± 0.3	−22.7	18.596 ± 0.614	3.3 %	4.09 ± 0.08
F2	84.13 ± 4.75	0.23 ± 0.02	−41.1	64.033 ± 0.252	0.39%	4.10 ± 0.01

### 3.5. Conductivity, dilution test, and pH measurement

#### 3.5.1. Conductivity test

To identify the type of ME, conductivity reading should be lower than 10 μS/cm in W/O type and 10–100 μS/cm in O/W type (Ngawhirunpat et al., 2013). Table 3 showed the conductivity were 18.596 ± 0.614 µS/cm (F1) and 64.033 ± 0.252 µS/cm (F2). The result indicated that both formulations were O/W type ME in nature with the presence of electro-conductive channels. Interestingly, F1 showed significantly lower electrical conductivity compared to F2 (*p* ≤ .05); although they contained the same amount of water (20%). This might be contributed to a higher content of ethanol in F2. Ethanol is an amphiphilic molecule that has a hydrophilic hydroxyl group allowing it to partition into the water, and a hydrophobic 2-carbon alkyl chain to facilitate the remobilization and redistribution of compounds in the aqueous phase. Note that pure ethanol has zero electrical conductivity because it does not contain any electrolytes. However, when it is used as a co-surfactant in ME, ethanol intercalates in the system and forms less rigid interfacial film between the water and oil phases, leading to complex molecular interactions to retain the oil droplet globules in the system and maintain the ionic mobility of the water (Szumała, 2015).

#### 3.5.2. Dilution test

The dilution test was performed to identify the type of ME formulation. For the tested ME, excess water was easier to be dispersed than oil, and more difficult to return to a single and clear phase with excess oil. This result confirmed that both F1 and F2 were O/W type ME. It is advantageous to incorporate ibuprofen into O/W type ME as the drug is protected in the internal oil phase as a barrier for oxygen diffusion, thus preventing oxidative degradation by the external aqueous phase.

#### 3.5.3. pH measurement

To minimize nasal irritation, drug products delivered through the nasal cavity are commonly adjusted to have pH values between 4.5 and 6. The mild acidity can also provide an environment for lysozymes found in nasal secretion to destroy certain bacteria. In alkaline surroundings, they are inactivated, and the nasal tissue is susceptible to microbial infection (Singh et al., 2012). Table 3 showed that the pH values were 4.09 ± 0.08 and 4.10 ± 0.01 in F1 and F2, respectively, which were very close to the recommended pH for nasal application; therefore, minimal nasal irritation would be expected. There was no significant difference between the pH of these two formulations (*p* > .05).

### 3.6. Rheological study

Viscosity characterizes the microscopic, macroscopic structures and their internal interaction in the ME system. Since a ME is formed with micrometer-sized oil and water droplets, it will have low viscosity if these droplets are not interacting in the system. On the contrary, higher viscosity of ME will be formed when the interaction of cylindrical and wormlike micelles is taking place in the process (Lawrence & Rees, 2000). One of the unique characters associated with ME is the presence of different internal structures which are classified by Winsor (Winsor, 1948) and each class has its distinct viscosity and rheological behavior (Alany et al., 2008). It is possible for a system that is oil continuous to change into a bicontinuous system and finally to a water continuous system.

**Figure 2.**
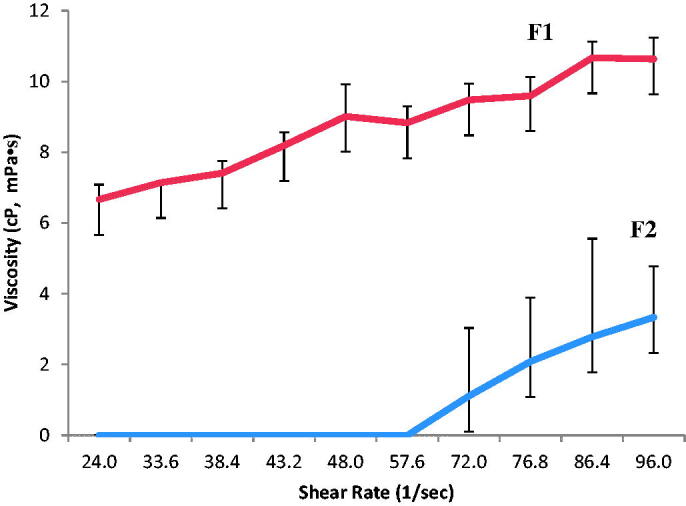
Viscosity of freshly prepared F1 and F2 as a function of shear rate at 25 °C (*n* = 3).

For intranasal ME application, low viscosity is preferred for good flowability, easier handling during the administration, and to avoid nasal mucociliary clearance. The viscosity as a function of shear rate was presented in Figure 2 which indicated low viscosity with 10.63 ± 0.60 cP in F1 and 3.33 ± 1.44 cP in F2 at a shear rate of 96. There was a minor increase in viscosity after speeding up the shear rate which could be attributed to the alteration in the internal structure due to the modification of droplets shape or conversion from O/W to bi-continuous ME under a higher shear rate (Mehta et al., 2010); however, these changes were slow.

**Figure 3.**
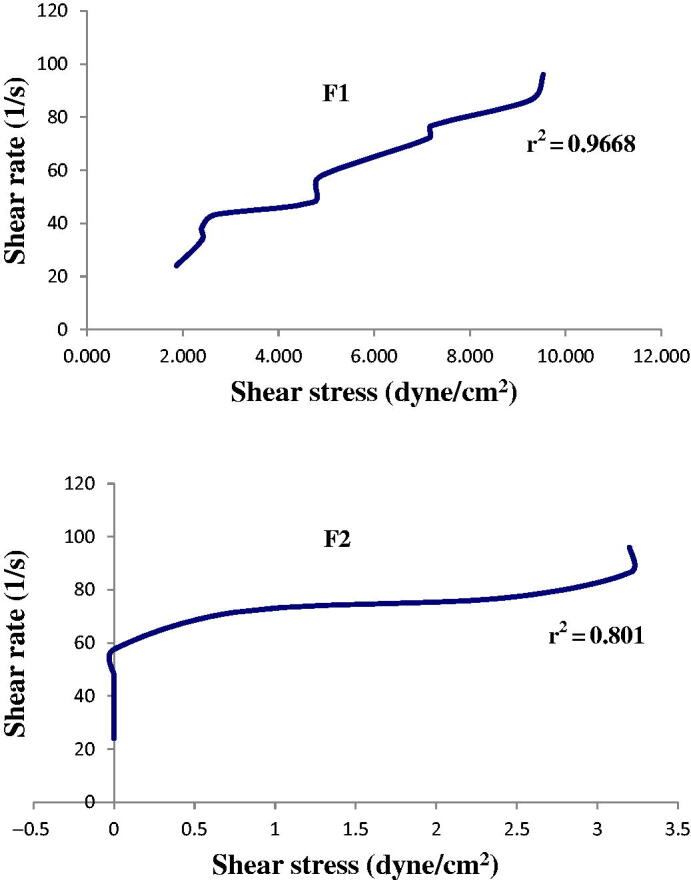
Rheogram of the freshly prepared F1 and F2 at 25 °C (*n* = 3).

Rheogram of the freshly prepared F1 and F2 in Figure 3 indicated that the slopes of the linear plots between shear rate and shear stress (*r*^2^) were 0.9668 and 0.801 for F1 and F2, respectively. F1 showed a Newtonian flow behavior with *r*^2^ >0.9 and F2 showed a non-Newtonian flow behavior and plasticity (Tashtoush et al., 2013). The rheological difference of these two formulations may be contributed to the fact that there is a change in the internal microstructure of F2 due to either the change in the shape of droplets or the transition from O/W to bi-continuous microemulsion (Moulik & Paul, 1998). The longer side chain of Tween 80 in F2 might be another factor for different interfacial packing compared to Tween 20 used in F1 (Mehta et al., 2010).

### 3.7. Transmission electron microscopy (TEM)

**Figure 4.**
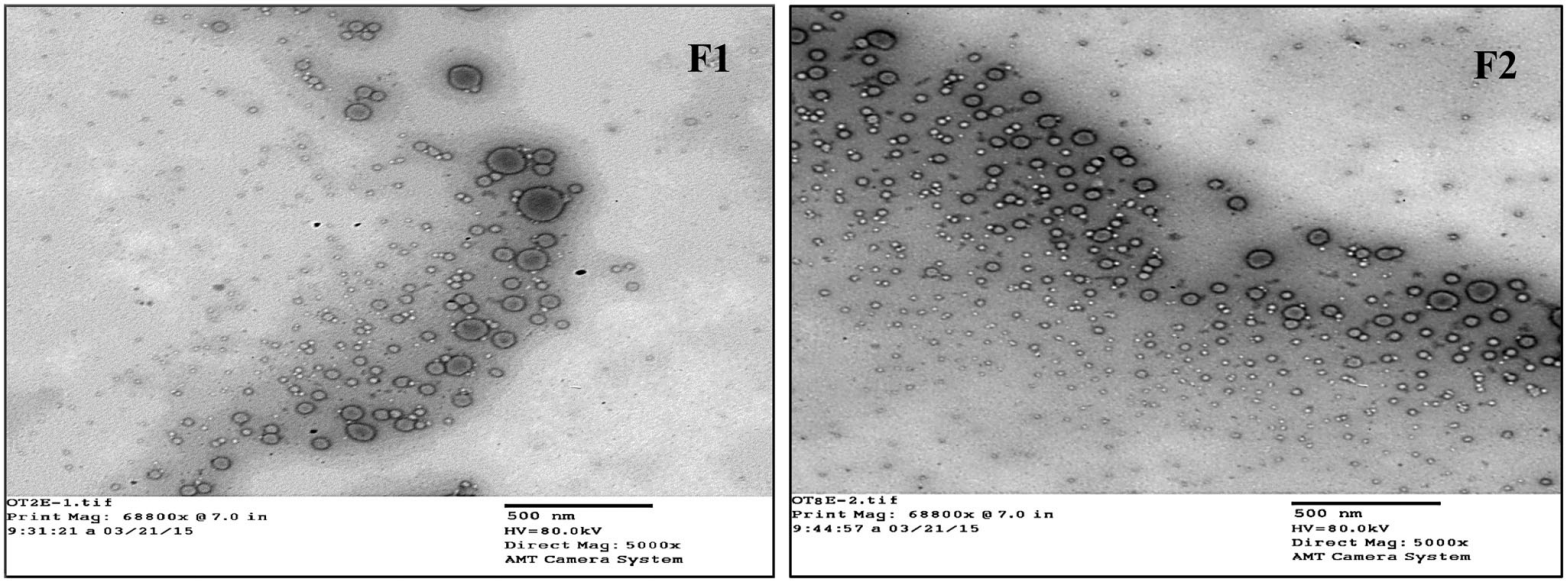
Transmission electron microscopic images of freshly prepared F1 and F2 formulations (Mag. 68800).

Figure 4 showed spherical-shaped droplets in the transmission electron microscopic images of both freshly prepared F1 and F2 formulations (Mag. 68800).

### 3.8. *In vitro* drug release study

The drug release from ME depends on various formulation parameters such as oil to aqueous phase ratio, droplet size, distribution of the drug in the phase system, and the rate of diffusion. We compared drug release from F1 and F2 to ibuprofen solution in propylene glycol to investigate the formulation excipient factor. Solubility study of ibuprofen in phosphate buffer solution pH 7.4 was 4.20 ± 0.055 mg/ml; accordingly, 100 µl of the tested formulations were placed in the donor compartment with ibuprofen concentration of 200 mg/ml to ensure sink condition in the receptor chamber throughout the experiment.

**Figure 5.**
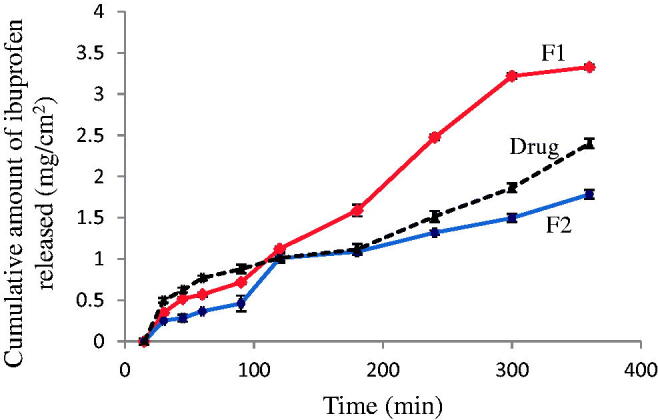
The release profiles of ibuprofen from F1, F2 and ibuprofen solution in propylene glycol, in phosphate buffer, (pH 7.4) at 34 ± 0.5 °C (*n* = 3).

Figure 5 represented ibuprofen release profiles over time showing a steady release with the cumulative amount of ibuprofen released from F1 of 3.38 ± 0.61 mg/cm^2^ and F2 of 2.43 ± 0.12 mg/cm^2^ at 6 h. Drug release from F2 was significantly lower compared to F1 (*p* ≤ .001), suggesting remarkable factors of the interactions between the drug and different phase components. Since the difference between F1 and F2 in the formulation is the ratio of *S*_mix_ and the type of surfactant, the divergent release profiles might be attributed to the active mobility of the drug within the vehicles in the formation of ME. This result agreed with data reported by Djordjevic et al. (Djordjevic et al., 2005) that the rate of drug release from ME depends on the vehicle, viscosity, and the existence of surfactant micelles. The release of ibuprofen from the existing interfacial film or embedded in the internal phase into the aqueous medium was prolonged by diffusion across the interfacial structure which was further affected by the types of oil and surfactants in the ME. Tween 20 (polyoxyethylene sorbitan monolaurate) and Tween 80 (polyoxyethylene sorbitan monooleate) are amphiphilic, nonionic surfactants composed of fatty acid esters and polyoxyethylene long chains. They are generally not a homogenous mixture of a single fatty acid ester but are a mixture of esters of the fatty acids (Ha et al., 2002). Both types of polysorbates have a common backbone and only differ in the structure of the fatty acid side chain with lauric acid in Tween 20 and oleic acid in Tween 80. The hydrocarbon chains of these surfactants provide the hydrophobic nature of the polysorbates; while the polyoxyethylene side chains contribute to the hydrophilic part. Therefore, Tween 80 has more affinity to ibuprofen due to its lipophilic nature, resulting in retardation of drug release. Tween 80 also possesses higher interfacial tension than Tween 20 (Kerwin, 2008). The release of the lipophilic ibuprofen will require more energy and time to cross the interfacial structure to be released into the aqueous medium. Furthermore, ibuprofen released from the two tested formulations may also be affected by the different solubilization power of *S*_mix_. According to the solubility study, ibuprofen had higher solubility in Tween 80 and Tween 80: ethanol (1:4) compared to Tween 20 and Tween 20: ethanol (1:1); therefore, the drug release in F2 was hindered. On the other hand, ibuprofen showed initial fast release in PG solution. Unlike ME, there was no oil core in the PG solution to hinder the release of ibuprofen. The release from PG solution became slower after 2 h compared to F1 which might be attributed to the higher viscosity of PG solution of 48.6 ± 0.13 cP compared to 10.63 ± 0.60 cP in F1 (Dow Chemicals, Propylene Glycol, 2014). The higher release of F1 is suitable for intranasal administration to provide rapid transport of drugs and avoid nasal mucociliary clearance.

### 3.9. *In vitro* nasal mucosa membrane permeation study

**Figure 6.**
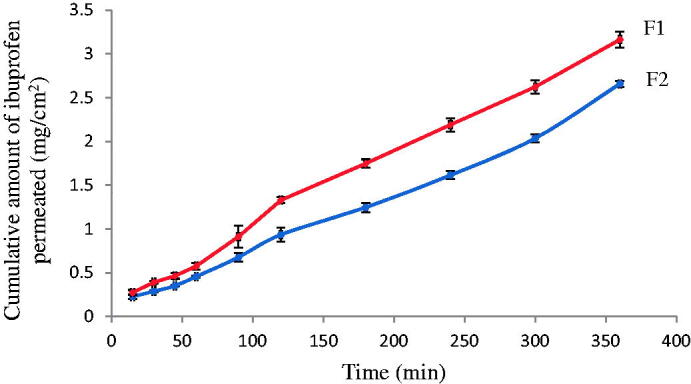
In vitro ibuprofen permeation profiles through sheep nasal mucosa from F1 and F2 in pH 7.4 phosphate buffer at 34 ± 0.5 °C (*n* = 3).

The amount of drug permeated through sheep nasal mucosa membrane of F1 and F2 were presented in Figure 6. Table 4 summarized the permeability parameters. The permeation profile showed a linear behavior in both F1 and F2. The amount of ibuprofen permeated from F1 was 3.03 ± 0.09 mg/cm^2^ that was significantly higher than F2 of 2.6 ± 0.03 mg/cm^2^ after 6 h (One way ANOVA analysis, *p* ≤ .001), demonstrating the remarkable effect of *S*_mix_ on the permeation of ibuprofen from the prepared ME. The higher ibuprofen solubility in *S*_mix_ (1:4) of F2 might potentially slow the drug release; consequently, lower the permeation through the nasal membrane. With the drug release of 3.38 ± 0.61 mg/cm^2^ and the permeation of 3.03 ± 0.09 mg/cm^2^, F1 demonstrated a 90% permeation rate from the released drug. Moreover, a longer lag time of 30.16 min in F2 compared to 21.9 min in F1, and a lower flux of 6.8 ± 1.83 µg/cm^2^/min in F2 compared to 8.4 ± 2.01 µg/cm^2^/min in F1 provided further evidence on the impact of vehicle influence in the nasal mucosa membrane permeation.

**Table 4.** Permeation parameters of ibuprofen through sheep nasal mucosa membrane from F1 and F2.

Formulation	Flux (µg/cm^2^/min)	Permeability coefficient (*K_P_*) (×10^−3^ cm/min)	Lag time (*T_L_*) (min)	Diffusion coefficient (*D*) (×10^−6^ cm^2^/min)
F1	8.4 ± 2.01	0.42	21.9	23.13
F2	6.8 ± 1.83	0.34	30.16	16.79

Olfactory mucosa in humans is present approximately in the upper 7 cm of the nostril. Nevertheless, there is no clear borderline between respiratory and olfactory mucosa (Perry et al., 2002). For a drug to reach the brain directly from the nasal cavity bypassing BBB, it has to reach the olfactory mucosa which is much thinner than the studied sheep nasal mucosa (Boron & Boulpaep, 2016), suggesting that even better permeation can be expected through the human olfactory pathway into the brain. In addition, the intranasal administration should be deeper into the nasal cavity to ensure olfactory targeting and avoid drug loss through permeation into the nasal mucosa along the way. Consequently, an ibuprofen-loaded F1 formulation was selected to continue the in vivo evaluation.

### 3.10. Stability study

#### 3.10.1. Accelerated stability study

**Figure 7.**
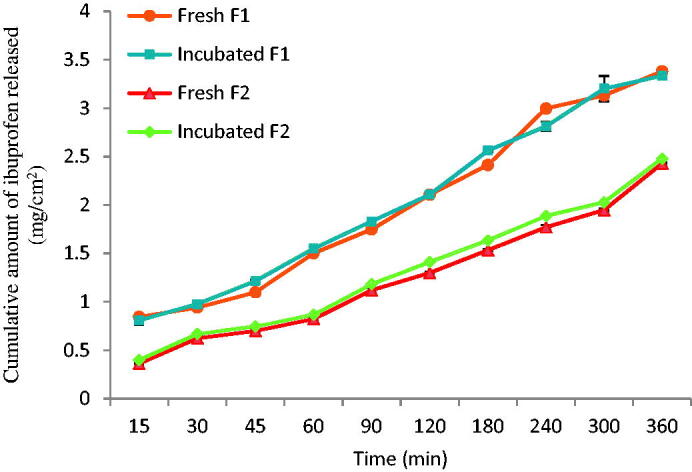
*In vitro* drug release profiles of ibuprofen from fresh and incubated F1 and F2 at 40 °C and 60% RH for 3 months (*n* = 3).

**Figure 8.**
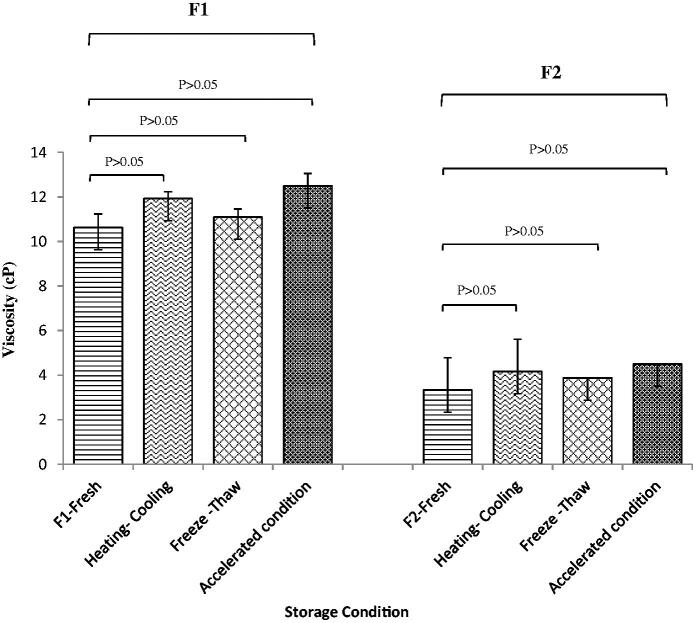
Viscosity at 96 shear rate of fresh, after freeze-thaw cycles, after heating-cooling cycles and after storage at accelerated condition of F1 and F2 (*n* = 3).

Investigated samples were periodically examined during the 3-month accelerated storage conditions (40 °C and 60% RH). No physical changes or drug precipitation was observed. Table 5 presented the comparison of drug content recovery, particle size, and pH between the fresh and stored samples. Statistical analysis indicated no difference between these stored samples and the fresh samples (One-way ANOVA, *n* = 3). The in vitro drug release through the cellophane membrane of these stored samples was also evaluated. The results in Figure 7 indicated that there was no significant difference in the amount of ibuprofen released from the freshly prepared F1 and F2 compared to the incubated F1 and F2. (*p* > .05), indicating the stability of both formulations under accelerated conditions. Further comparison of viscosity of stored F1 and F2 at 96 s^−1^ with the fresh samples of F1 and F2 also showed no significant difference (*p* > .05) (Figure 8).

**Table 5.** Drug content recovery, particle size, and pH of fresh and stored samples at 40 °C and 60% RH for 3 months (*n* = 3).

	F1			F2		
	Fresh sample	Accelerated stability sample	One-way ANOVA *p*-value	Fresh sample	Accelerated stability sample	One-way ANOVA *p*-value
Drug content recovery %	98.74 ± 3.58	99.66 ± 2.19	*p* > .05	99.45 ± 3.9	97.99 ± 0.5	*p* > .05
Particle size Mean ± SD (nm)	166.3 ± 2.55	171 ± 2.32	*p* > .05	84.13 ± 4.75	90.34 ± 3.32	*p* > .05
pH	4.09 ± 0.08	3.96 ± 0.12	*p* > .05	4.10 ± 0.01	4.18 ± 0.12	*p* > .05

#### 3.10.2. Physical stability study

##### 3.10.2.1. Heating-cooling cycle

Following six heating-cooling cycles, no change was observed in the physical appearance of F1 and F2 in terms of transparency, phase separation, or drug precipitation. Both F1 and F2 were considered physically stable under stressed heat and cool cycles. The viscosity of F1 and F2 also showed no difference compared to the fresh sample (*p* > .05) (Figure 8).

##### 3.10.2.2. Freeze-thaw cycle

After three freeze-thaw cycles, the physical appearances of F1 and F2 were unchanged; neither drug precipitation nor phase separation was detected. Under the stress at a temperature below the freezing point, the formation of ice crystals might cause oil droplets to elongate and flatten in O/W type ME. Besides, the lipophilic portion of the surfactant could lose its mobility; while the hydrophilic portion would likely dehydrate due to the freezing action of water. After thawing, water is liberated and travels through the ME. In a stable ME, the system could heal itself before coalescence occurs to survive the test (Jain et al., 2010). The viscosity of F1 and F2 after the freeze-thaw test showed no difference compared to the fresh sample (*p* > .05) (Figure 8), indicating that the recovering of the system was quickly taking place after the freeze-thaw stress.

##### 3.10.2.3. Accelerated centrifugation test

The accelerated centrifugation test showed good physical stability after centrifugation without phase separation, drug precipitation, or change of clarity in F1 and F2.

#### 3.10.3. Long term stability study

To simulate the real product storage condition, F1 and F2 were stored under ambient temperature for 6 and 12 months. Periodically samples were examined for their appearance which showed no signs of drug precipitation or phase separation, and no color change during the 12-month storage. Table 6 showed the drug content recovery and pH of the stored samples compared to the fresh formulations. There was no statistically significant difference between the fresh and stored samples in 6-month and 12-month in F1 and F2 (*p* > .05).

**Table 6.** Drug content recovery and pH of fresh F1 and F2 formulations and after storage for 6 and 12 months at room temperature (40% humidity) (*n* = 3).

	F1		F2	
	Drug content recovery (%)	pH	Drug content recovery (%)	pH
Fresh prepared	98.74 ± 3.58	4.09 ± 0.08	99.45 ± 3.9	4.10 ± 0.01
Stored for 6 months	98.50 ± 1.37	4.12 ± 0.09	97.82 ± 1.9	4.15 ± 0.12
Stored for 12 months	97.82 ± 2.11	3.98 ± 0.13	98.98 ± 1.8	4.17 ± 0.09

### 3.11. *In vivo* study

Under applied HPLC chromatographic conditions in the brain homogenate assay, ibuprofen and ketoprofen were well separated with retention times of 3.3 and 5.7 min, respectively. No interfering peaks from endogenous substances in the brain homogenate were detected in the chromatogram. A linear relationship was confirmed over the concentration range of 0.05–1 μg/ml (*r*^2^ = 0.999). The linear relation obeyed Beer-Lambert’s law. All CV % values for the intra-day, inter-day precision and accuracy, and repeatability data ranged 1.33–9.52%, 0.653–3.468%, and 0.303–7.709%, respectively, indicating high precision, accuracy, and repeatability with CV% less than 10%. The percentage of ibuprofen recovery ranged 95.319–105.02% in inter-day and 94.89–104.428% in intra-day, respectively.

**Figure 9.**
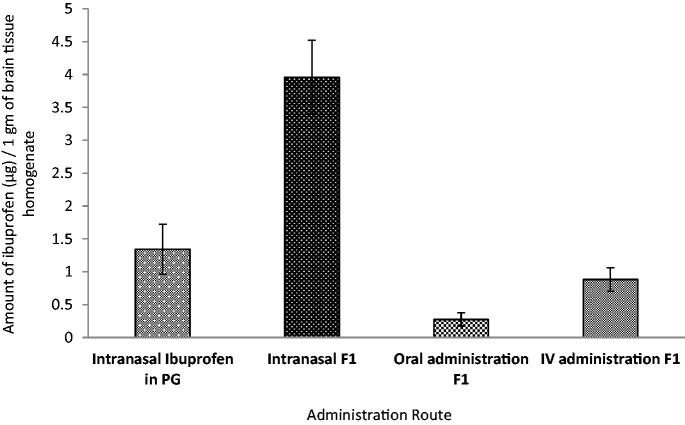
The amount of ibuprofen recovered in 1 g of brain tissue homogenate following intranasal administration of the reference ibuprofen in propylene glycol solution and F1 compared to oral and intravenous administration of F1in rats (*n* = 3).

Figure 9 showed that the brain uptake of ibuprofen was almost four times higher in F1 compared to the reference of ibuprofen in PG solution, and this difference was statistically significant (*p* ≤ .001). The choice of PG as a solvent was based on both the solubility study data as well as the fact that it was not reported to have any influence on the membrane permeation (Turner et al., 2011).

The distribution of human olfactory mucosa in the nasal cavities extends through the upper and middle turbinates (Escada, 2013), which is different from the respiratory mucosa that covers most of the surface of the nasal cavity. To achieve brain targeting, drug administration should be deep into the nasal cavity to prevent drug absorption through respiratory mucosa into the systemic circulation and ensure that the drug has a higher chance to reach the olfactory region. In general, drugs can be delivered by a transcellular pathway or paracellular route through the tight junctions between the cells in which polar drugs with molecular weights below 1000 Da commonly enter (Illum, 2003). Ibuprofen is an acidic compound with a pKa of 4.43 ± 0.03 (Schettler et al., 2001); hence, at nasal mucosa pH (pH 5.5), there should be about 12% of ibuprofen remains unionized. Therefore, the transport of ibuprofen through the transcellular pathway is very low. Consequently, the brain uptake of ibuprofen is mainly transported via the paracellular pathway. The significant difference in brain uptake of ibuprofen between F1 and the ibuprofen in propylene glycol solution further confirmed the importance of formulation factors on the impact of efficacy and performance of intranasal delivery. These formulation factors should be considered while designing the drug delivery system for intranasal administration to achieve a rapid transport of drug across olfactory mucosa. F1 ME showed the potential as a candidate for intranasal delivery.

The amount of brain uptake of ibuprofen through intranasal administration was also compared to oral and intravenous administrations (Figure 9). The concentration reached the brain from both intranasal administration of ibuprofen solution and ME were higher than IV and oral administration. When drug concentration in the brain is significantly higher through intranasal administration than that of IV or oral administration, a direct pathway from the nasal olfactory region to the brain is postulated to be the predominant route. In general, lipophilic drugs are absorbed transcellular across the nasal membrane, presenting similar pharmacokinetic profiles to those obtained after IV administration (Bitter et al., 2011). The amount of ibuprofen reaching the brain through intranasal administration of F1 ME demonstrated nearly 10-fold and 4-fold higher compared to oral and IV administration, respectively. This result presented evidence of a promising formulation factor of ibuprofen ME (F1) for brain targeting through olfactory transportation. Furthermore, the oil and *S*_mix_ components in F1 might also act as penetration enhancers on the tight junctions and/or function as enzymatic inhibitors in the nasal cavity to reduce the chance for enzymatic degradation in the nose (Singh et al., 2012).

Although many novel nasal products for systemic treatment of diseases are present in the market, there are no marketed products approved yet using the intranasal route for brain targeting to treat CNS diseases such as AD. The development of drug delivery in this domain, enabling a rapid pathway and sufficient drug concentrations to the brain, represents an urgent challenge.

### 3.1. Nasal ciliotoxicity study

The nasal mucosa is characterized predominantly by a pseudo-stratified ciliated columnar epithelium (Kia’I and Bajaj, 2019). The mucous film on the respiratory epithelium is continually moved by ciliary action. This mucociliary clearance mechanism helps to propel all hazardous organisms such as dust, irritants, microorganisms as well as carcinogenic substances and provides a primary nonspecific defense mechanism against foreign substances entering the human body. Therefore, to consider using the intranasal route for drug delivery, the formulation should not adversely disturb or damage the existing mucociliary clearance system. It is crucial to examine the influence of the drug and excipients on the nasal mucociliary system before clinical application of the investigated formulation to guarantee its safety.

**Figure 10.**
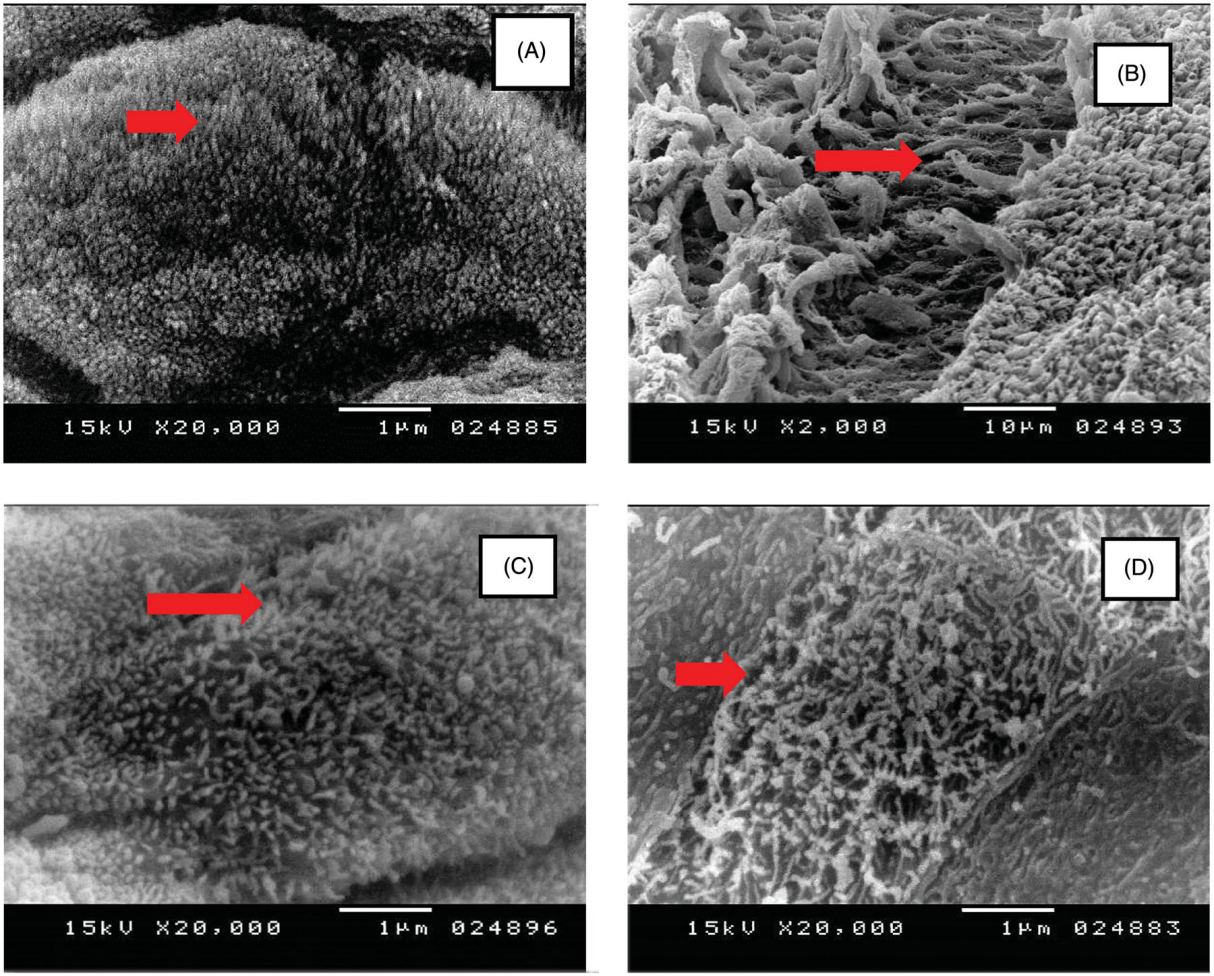
Scanning electron microscopic images of the rat nasal cilia treated with: (A) Normal Saline (negative control), (B) Isopropyl Alcohol (positive control) microemulsion, (C) Blank F1 (without ibuprofen), (D) Ibuprofen-loaded F1.

SEM images of the histological changes after intranasal administration of F1 ME compared to normal saline (negative control), isopropyl alcohol (positive control), and blank F1 were shown in Figure 10. Nasal mucosa treated with normal saline (Figure 10(A)) showed an intact epithelium layer without any damage which was contrary to the nasal mucosa treated with isopropyl alcohol (Figure 10(B)) showing extensive damage of the epithelium layer into the deeper tissue portions coupled with the loss of nasal cilia. The remaining cilia had an irregular appearance of the ultrastructure. The image of the nasal mucosa treated with blank F1 (Figure 10(C)) showed no damage to the epithelial layer and other parts of the mucosa, suggesting the safety of the excipients used in the formulation with no toxic effects on the rat nasal mucosa. Figure 10(D), the nasal mucosa treated with F1 ME, showed no mucociliary damage and no marked effect on the length, density, and ultrastructure of the cilia. The appearance of the cilia and the epithelial layer was intact. These results are in congruence with the findings of many other related studies reporting that drug-loaded micro/nanoemulsions are not ciliotoxic and safe for intranasal administration (Haider et al., 2018; Kia’I and Bajaj, 2019).

## 4. Conclusions

Although ibuprofen had been on the market for a long time, it had not been tested through intranasal administration for brain targeting. In this study, the selected ibuprofen ME formulation (F1) demonstrated constant colloidal dispersion properties, appropriate drug release, and excellent stability upon exposure to various stressful stability tests. The promising nasal mucosal permeation and suitable viscosity of F1 allowed more amount of ibuprofen to reach the brain through the olfactory pathway. *In vivo* animal study further showed higher drug concentration reaching the brain through intranasal administration compared to intravenous and oral routes. The studied formulation F1 assured safety for intranasal administration with no nasal ciliotoxicity compared to the positive and negative control, and blank formulation. To reduce mucociliary clearance when dosing repeated regularly in the nose, many strategies could be applied in further studies, such as the incorporation of bioadhesive carrier, adjustment of the viscosity of the formulation, and using an application of a novel administration device. The present study demonstrated a great repurposing potential using ME as a dosage form to deliver ibuprofen for brain targeting through intranasal administration for the management of Alzheimer’s disease.
